# Impact of environmental contaminants in fish on cell death and oxidative stress using *in vivo, in vitro*, and molecular docking

**DOI:** 10.29219/fnr.v69.12687

**Published:** 2025-10-28

**Authors:** Saber Abdelkader Saïdi, Othman Ahmed Alghamdi, Mohiuddin Khan Warsi, ElFeki Abdelfattah, Jos van Pelt

**Affiliations:** 1 Department of Biological Sciences, College of Science, University of Jeddah, Jeddah, Saudi Arabia; 2 Animal Ecophysiology Laboratory, University of Sfax, Sfax, Tunisia; 3 Laboratory of Clinical Digestive Oncology, Department of Oncology, KU Leuven & University Hospitals Leuven and Leuven Cancer Institute (LKI), Leuven, Belgium

**Keywords:** *fish products*, *food contaminants*, *oxidative stress*, molecular docking, iron toxicity, in silico study

## Abstract

**Background:**

Fish species from the Red Sea constitute excellent food sources but may be unsafe to consume because their bioaccumulation of iron (Fe) is greater than the recommended concentration.

**Objective:**

We investigated the safety concerns related to the consumption of fish containing iron.

**Design:**

In this study, Wistar rats were treated with Fe(II) and Fe(III) at a total dose of ~200 mg/kg body weight. For cytotoxicity testing, human liver WRL-68, human hepatoma HepG2, and rat liver FTO2B cells were exposed to Fe(II) and Fe(III). Computational tools were utilized to assess the molecular interactions of iron with critical oxidative stress markers and predict potential toxicological outcomes.

**Results:**

The in vivo results showed that only treatment with Fe2+ significantly (*P* < 0.05) changed aminotransferase activity compared to the control and caused an alteration in the oxidative balance, which was reflected by increases in the content of malondialdehyde (MDA) and the activities of antioxidant enzymes. The in vitro results revealed that the concentrations of Fe(II) and Fe(III) typically found in Red Sea fish were not toxic to these cell lines. However, the addition of Fe(III) potentiated the harmful effects of H2O2 in FTO2B cells. Moreover, the results demonstrate that exposure to Fe(III) resulted in increased (*P* < 0.05) expression of the superoxide dismutase (SOD) gene. The docking analysis revealed that Fe^2+^-Protoporphyrin exhibited strong binding to both SOD (–8.7 kcal/mol) and Catalase (–9.1 kcal/mol), as compared to its known inhibitors, suggesting a potential role in enzymatic inhibition and oxidative stress modulation.

**Conclusion:**

The results reveal that there is a potential risk of toxicity when fish products are consumed. Further investigations are needed, especially in regard to determining the estimated weekly intake of these metals.

## Popular scientific summary

- This study indicated that cells must be stressed after consuming moderate amounts of Fe(II) and Fe(III) from fish products.
- The docking analysis revealed that Fe^2+^-Protoporphyrin exhibited strong binding to both SOD and CAT, suggesting a potential role in enzymatic inhibition and oxidative stress modulation.
- Thus, information on iron and/or metal concentrations in fish and sea products in general is important to assess the possible exposure of the community to toxic compounds after their consumption.

Biological activities require iron (Fe), but it is extremely toxic when present in excess (1, 2). The two main kinds of available Fe are Fe(II) in reducing intracellular environments and Fe(III) in aerobic environments (3). Iron is a redox-active metal that controls cell development and differentiation as well as oxidation–reduction reactions. It acts as a prosthetic group for several proteins involved in essential cellular functions, including ribonucleotide reductases and DNA polymerases during DNA synthesis, cytochromes in the electron transport chain for cellular respiration, hemoglobin and myoglobin for oxygen transport and energy generation, and numerous enzymatic reactions (4–9). Many proteins and enzymes that maintain a variety of physiological activities contain iron as an essential component (10, 11). Despite the fact that iron is a trace metal present in the diet, anemia affects more than 2 billion people worldwide (12–15). Additionally, iron can be toxic in excess due to its capacity to stimulate the generation of hazardous reactive free radicals via the Fenton reaction (16–18).

Iron is most commonly stored the liver, and iron can seriously damage other organs (19, 20). In addition to its role in detoxification, the liver is essential for the metabolism of biological molecules. Most substances that are absorbed pass through the intestinal barrier and enter the liver, where toxic elements can accumulate. When studying the effects of pollution, the liver is the most vital organ to take into consideration (21). Iron accumulation in hepatic tissues is a secondary effect of frequent blood donation and noticed in patients with long-term liver disorders, such as alcohol-related liver disorders and persistent viral hepatitis (22–24). Recent research has revealed that the physiopathology of Fe-induced hepatic damage and cell death may involve oxidative stress, which is facilitated by free radicals and reactive oxygen species (ROS) (25–28).

The Red Sea receives discharge of a significant amount of wastewater from the Jeddah Metropolitan Area daily (29, 30). Each day, this area receives 100,000 m^3^ of sewage sludge, which lowers the water quality, increases human exposure and ecological and health risks, and causes an overall rise in the mortality of aquatic species (29, 31). Indeed, heavy metals are regarded as important classes of toxic substances that penetrate aquatic systems by anthropogenic processes and/or atmospheric discharge in addition to organic compounds. Aquatic systems are contaminated with significant quantities of heavy metals, which accumulate and become amplified in sea water, sediment, and the food chain, eventually posing a major threat to human health (21, 32). In fact, the average amount of Fe discovered in the liver of the fish species *Variola louti* from the contaminated region was 4020.01 µg/g liver tissue (30). Additionally, according to a recent study, the concentration of iron in the tissues of economically important species of marine fish collected from the coastal region of Jeddah ranged from 81.60 to 188.60 mg/kg dry weight (33).

In a prior study, oxidative stress caused by iron overload produced excessive ROS generation and led to serious liver damage (34). However, there is insufficient knowledge regarding the use of Fe(II) or Fe(III) as a potential preventive agent against cell injury induced by oxidative stress. Furthermore, it has been recognized that organisms have enzyme-based antioxidant defenses, such as superoxide dismutase (SOD) and catalase (CAT), that are essential for preserving cellular homeostasis by eliminating ROS (35, 36). Recent research has increasingly focused on the regulation of antioxidant gene expression as a potential indicator of oxidative stress.

Thus, evaluating the effects of Fe(II) and Fe(III) *in vivo* and *in vitro* may help to better understand the toxicity or benefits of iron and to evaluate the potential health effects. Therefore, the current study was performed to explore the impact of Fe(II) and Fe(III) on cell death and oxidative stress using human and animal liver cell lines and an in vivo rat model. To assess the possible intensification of iron cytotoxicity that occurs via oxidative stress, the toxicity of H_2_O_2_ combined with iron to liver cells was investigated to imitate reactive oxygen species generation by the body. We conduct also an in silico evaluation of the molecular docking of iron on key oxidative stress-related proteins, and we analyzed the interactions of Fe^2+^-Protoporphyrin, Diethyldithiocarbamate, and Amitrole with oxidative stress-related enzymes, SOD and CAT.

## Materials and methods

### Solutions

FeSO_4_ (215422, Sigma) and FeCl_3_ (701122, Sigma) (50 mM final concentration) were added to sterile H_2_O to make iron stock solutions. Each day, fresh solutions of Fe(II) and Fe(III) were prepared. The stock solution of H_2_O_2_ (H1009, Sigma) was 9.79 M.

### Animals and experimental design

This study included 30 male Wistar rats weighing 150–200 g who were chosen randomly. The Local Ethics Committee on Animal Experiments approved the experimental procedures (ethics approval number: 1204). The animals were distributed randomly into three groups (*n* = 8), and they were given a commercially available low-iron diet (5 mg/Kg diet) that contain corn, soya, and vitamins (Table 1). Rats in the Control group (Control) were fed low-iron diet without injection of iron, while rats in the Fe(II)- and Fe(III)-treated groups were given iron intraperitoneally (i.p.) once per week for 4 weeks for a total dose of 200 mg/kg body weight. The concentrations were chosen based on the results published by Younis et al. (33), where the iron concentration in the muscle (consumable part) of different commercial and high nutritive value marine fish species from the Jeddah Coast was determined. At the end of the 2-week study period, the animals were anesthetized with pentobarbital (Y0002194, Nembutal, 50 mg/kg), and blood was withdrawn from the abdominal aorta for liver function and oxidative stress parameter analysis. The rats were then sacrificed by exsanguination under anesthesia. Liver samples were collected and fixed in a 10% formaldehyde solution before being stored in 70% ethanol for histological analysis.

**Table 1:** Composition of rat diet: low-iron diet contains corn, soya, vitamins, and minerals.

Ingredient	Low-Iron Diet (LID)
Nutritional properties (%)	
Moisture	14
Fibers	3.4
Proteins	22
Lipids	3.5
Ash	6.7
Carbohydrate	50.4
Caloric value (kcal/kg)	2,850
Amino acids (%)	
Methionine	60
Cysteine	0.38
Threonine	0.80
Tryptophane	0.30
Mineral mix (mg/kg)	
Manganese	80
Iron	5
Copper	18.75
Zinc	65
Selenium	0.30
Cobalt	0.20
Iode	1.20
Vitamins and antioxidants (mg/kg)	
Vitamin A	13,000
Vitamin D3	4,375
Vitamin H	62.5
Antioxidants (BHA-BHT)	125

### Liver function

The levels of serum aminotransferases were used as established markers of hepatic injury. Alanine aminotransferase (ALT) was measured using the glutamic-pyruvic transaminase (GPT) Alanine Aminotransferase (ALAT) IFCC mod. kit produced by Human Gesellschaft für Biochemica und Diagnostica mbH, Germany, according to Reichling and Kaplan (37). As stated by Schumann and Klauke (38), aspartate aminotransferase (AST) was determined using the glutamate oxaloacetic transaminase (GOT) Aspartate Aminotransferase (ASAT) IFCC mod. kit produced by Human Gesellschaft für Biochemica und Diagnostica mbH, Germany. Alkaline phosphatase (ALP) was estimated using the DEA Buffer, DGKC kit from Human Gesellschaft für Biochemica und Diagnostica mbH, Germany (39).

### Oxidative stress parameters

Investigations of thiobarbituric acid reactive substances (TBARS, Item No. 10009055), catalase (CAT, Item No. 707002), and superoxide dismutase (SOD, Item No. 706002) can reveal evidence of oxidative stress. These analyses were carried out using a commercially available IBL kit (IBL International, Hamburg, Germany), according to the manufacturer’s recommendations.

### Liver histology

Fixed tissue fragments were processed, embedded in paraffin for histological examination and cut to a thickness of 5 µm before collection and staining with hematoxylin and eosin.

### Cell culture

Hepatic cell lines (rat FTO2B cells, human WRL-68 cells, and human HepG2 cells) were used in this study. Stock solutions of FeSO_4_ (50 mM) and FeCl_3_ (50 mM) were prepared in sterile H_2_O. The cells were cultivated in 75 cm^2^ flasks, and trypsinization and counting were performed when the cultures were close to confluence. After counting, the cells were placed in 24-well plates (100,000 cells/well in 1 mL of medium). The medium was removed after 24 h and replaced with medium containing varying concentrations of iron and H_2_O_2_. In the samples without Fe(II) and Fe(III), equal volumes of media were supplied to cells to serve as controls. After an additional 72 h, the medium was removed and replaced with medium containing XTT solution (11465015001, Sigma), and the number of cells was counted. Fe^2+^ and Fe^3+^ stock solutions at five concentrations (2,500, 1000, 200, 100, and 50 µM) were employed in the cytotoxicity assay. An iron concentration of 100 µM was used in combination with H_2_O_2_. These concentrations were chosen based on the results published by Younis et al. (33), in which the iron concentrations in the muscle (consumable part) of five major marine fish from Jeddah Coast, Red Sea, were estimated.

### Cytotoxicity assay

After 72 h of culture in iron-containing medium, 500 µL of XTT solution was used to replace the medium. After placing the cells in a CO_2_ incubator for 4 h, the transformation of XTT into soluble formazan was determined using a plate reader. To compare cell viability percentages, cells that had not been exposed to iron or H_2_O_2_ in the same experiment were employed as controls.

### mRNA expression determined by RT-PCR

To gain more knowledge about the expression of genes (CAT and SOD) linked to oxidative stress, rat liver cells (FTO2B) were utilized. The cells were grown in the presence of Fe^3+^ (100 µM) and H_2_O_2_ (880 µM) to assess gene expression. Four replicates of each condition were used. After 72 h of exposure, TRIzol reagent was used to extract total RNA from the collected cells. In brief, chloroform (200 µL) was added to the sample tubes after 1 mL of TRIzol reagent (T9424, Sigma) was used to lyse cells. The same tubes were then centrifuged at 16,000 × g for 25 min at 4°C. After being transferred to different tubes, the aqueous phase was precipitated with ethanol (70%). In the presence of RNase-free water, the samples were centrifuged at 9,000 × g for 1 min to form RNA pellets. Then, the RNA was stored at –80°C. The 260/280 nm ratio was used to assess the RNA purity. Using reverse transcriptase, total RNA (2.5 g) was converted to cDNA. mRNA expression was amplified for 35 cycles by PCR using specific primers for SOD and CAT: SOD forward: 5′acaggattaactgaaggcgagcatggg3′, SOD reverse: 5′ccacaccgtcctttccagcagcc3′; CAT forward: 5′cccacgatattaccagatactccaaggc3′, CAT reverse: 5′agtttgccaactggtataagagggtagtcc3′; GAPDH forward: 5′tcatcatctccgccccttccgc3′, and GAPDH reverse: 5′aggcggcatgtcagatccacaacg3′. PCR was used to determine the expression of the genes encoding the enzymes glyceraldehyde-3-phosphate dehydrogenase (GAPDH), SOD, and CAT. As determined by PCR, the SOD, CAT, and GAPDH genes had sizes of 177, 684, and 390 bp, respectively. The SOD, CAT, and GAPDH pixel values were calculated using the Un-Scan-it V6.2 program, after which the agarose gel was digitally photographed. The proportions of CAT and SOD to GAPDH were considered.

### Data collection and molecular docking analysis

Structural data of oxidative stress-related proteins Superoxide Dismutase (SOD, PDB ID: 2C9V) and Catalase (PDB ID: 1DGF) were obtained from the Protein Data Bank (PDB). Heme iron, Fe^2+^-Protoporphyrin, PubChem CID: 4971 and two inhibitors Diethyldithiocarbamate, DETC: PubChem CID: 8987 and Amitrole, 3-Amino-1,2,4-triazole (3-AT): PubChem CID: 5517 were obtained from the PUBCHE Database. Molecular docking studies were performed using computational tools to predict iron-binding sites and affinities using Insta dock, PyMOL, and discovery studio.

Molecular docking was conducted with InstaDock (v1.0), a user-friendly docking tool that is running (version 1.1 of QuickVina-W) as its core engine (40). Protein-ligand docking was performed using the grid box with the center box lying at *X* = 20.321°, Y = 41.524°, and *Z* = 42.604, and the box size had reached a massive (Grid box dimensions) 103×112×120Å. A blind docking process covering whole protein surface can be undertaken. 9 poses were generated for each ligand, and the best pose (the smallest binding energy value) was selected for analysis. Protein structures are retrieved from the Protein Data Bank (PDB). They use the following PDB IDs: [1DGF]. The visualization was all done in BIOVIA Discovery Studio Visualizer (v2021) (BIOVIA, Dassault Systèmes; Discovery Studio Visualizer, Release 2021; San Diego: Dassault Systèmes, 2021).

### Analysis of the results

The results are presented as mean ± standard deviation (SD) of at least four separate trials, each of which was performed in duplicate. To perform the statistical comparisons, one-way analysis of variance (ANOVA) followed by Fisher’s Least Significant Differences test (LSD-test) was employed. *P* < 0.05 was considered to indicate statistical significance.

## Results

### Liver function

**Fig. 1:**
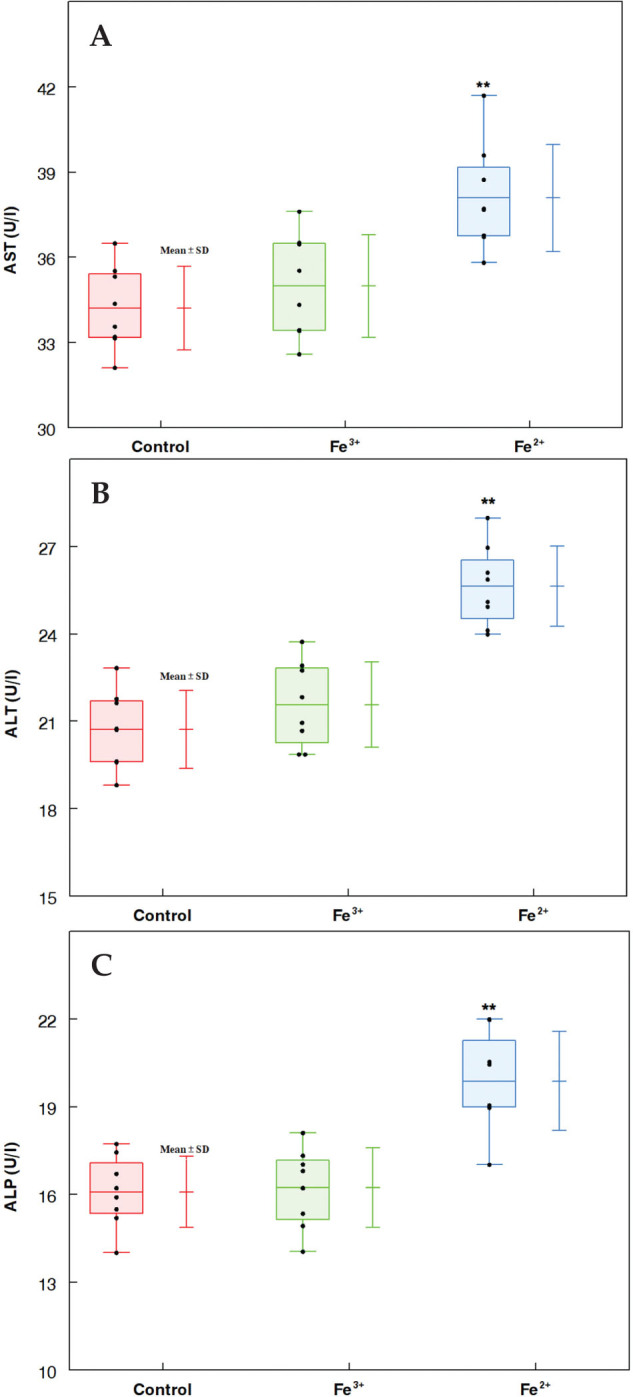
Biochemical markers in male rats following treatments with Fe(II) and Fe(III). Data are shown as mean ± SD. (A) Aspartate aminotransferase activity (AST); (B) Alanine aminotransferase activity (ALT); (C) Alkaline phosphatase activity (ALP). Using one-way ANOVA, *P < 0.05 and **P < 0.01 compared to the control.

At the end of the experiment and following exposure to iron, all of the rats survived with no signs of systemic toxicity. Figure 1 displays the biochemical parameters of liver function. When compared to the control group, the Fe^3+^ concentration had no effect on ALT, AST, or ALP activities. In contrast, enzyme activity was significantly augmented in the Fe^2+^ group compared to the control (*P* < 0.01).

### Effects of Fe^2+^ and Fe^3+^ on oxidative stress parameters

**Fig. 2:**
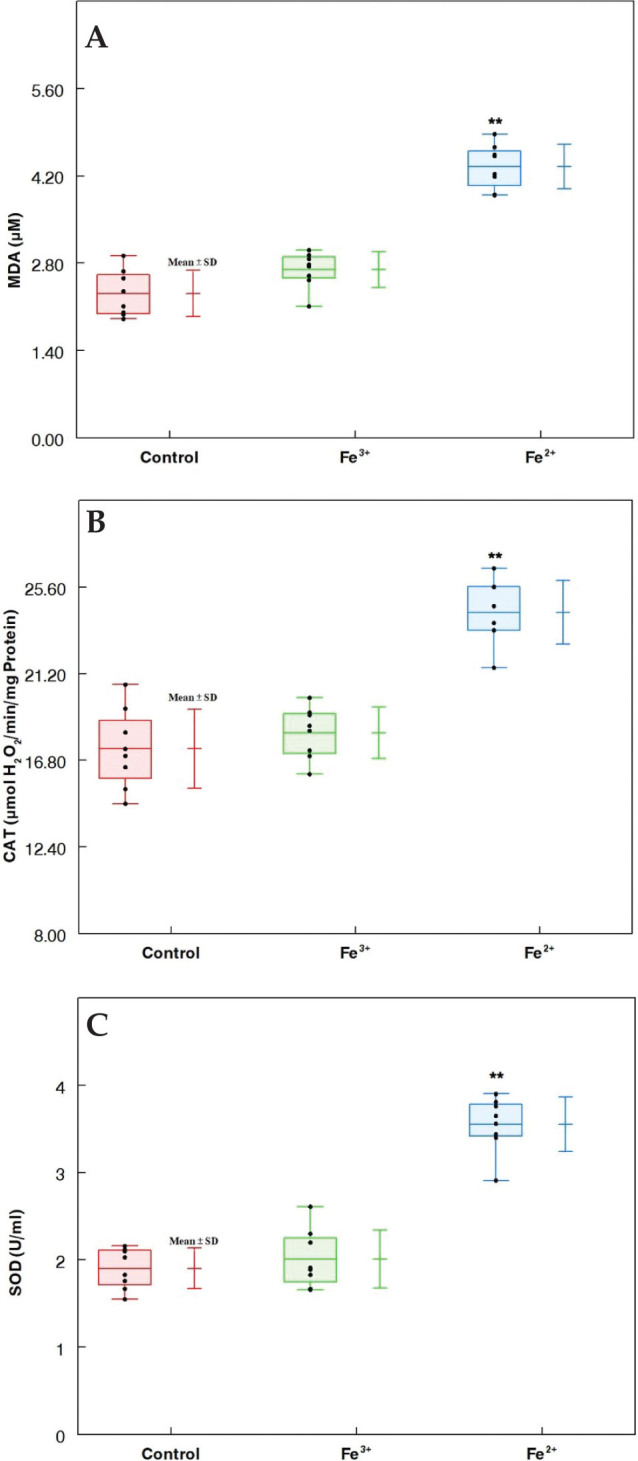
Oxidative stress parameters in male rats following treatments with Fe(II) and Fe(III). (A) MDA level, (B) CAT activity, and (C) SOD activity. Results are presented as mean ± SD. Using one-way ANOVA, *P < 0.05 and **P < 0.01 compared to the control.

Lipid peroxidation was assessed by examining the MDA levels in animal liver tissues. Fe^2+^ treatment produced a significant increase in MDA content in rat liver compared to the control (*P* < 0.01, Figure 2A). In the Fe^3+^ group, SOD and CAT activities were normal compared to those in the control group. In contrast, the Fe^2+^ group showed significantly increased levels of SOD and CAT compared with the control groups (*P* < 0.05, Figure 2B and C).

### Histology

**Fig. 3:**
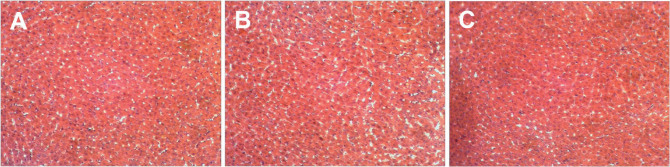
Liver histology from different treatment groups. Hematoxylin-eosin stain showing a normal structure of liver tissues in the different groups. (A) Control group, (B) Fe(II)-treated group, and (C) Fe(III)-treated group (magnification x 200).

Hematoxylin-eosin staining showed that the hepatic tissues had normal structures in the different rat groups, suggesting that the concentrations of Fe^2+^ and Fe^3+^ used in this study did not affect these tissues (Figure 3).

### Effect of Fe(II) on cellular viability

**Fig. 4:**
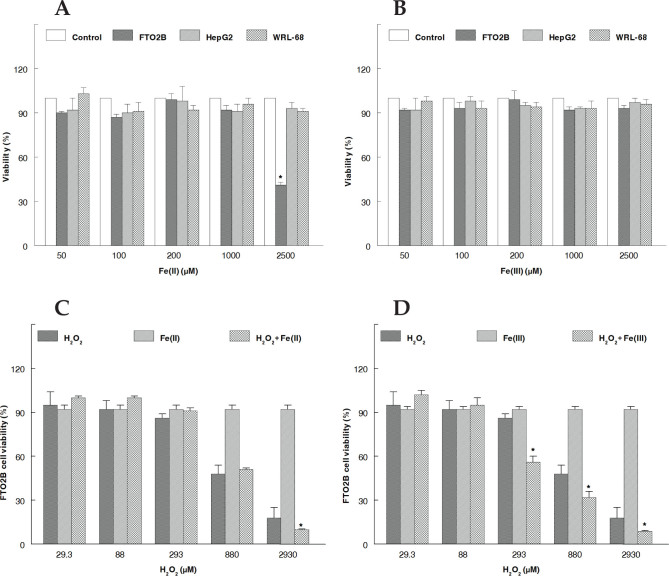
Impact of Fe(II) (A), Fe(III) (B), H_2_O_2_ (C), and H_2_O_2_ in cotreatment with Fe(III) (D) on hepatic cell lines viability determined after XTT incubation. Cell viability of the control was taken to be 100% at the specified concentration. The results are given as percentage, and every value represents mean ± SD. Using one-way ANOVA, *P < 0.05 and **P < 0.01 compared to the control. Viability (%): OD/OD_control_.

Figure 4A illustrates the cytotoxicity results measured by XTT assay in the three cell lines used after 72 h of Fe^2+^ stimulation. Treatment of FTO2B cells with Fe(II) resulted in a concentration-dependent loss in cell viability. Fe(II) was discovered to be cytotoxic to FTO2B cells at a concentration of 2,500 µM. The other cell lines were not damaged by the various Fe(II) concentrations.

### Effect of Fe(III) on cell viability

The cytotoxicity test showed that Fe(III) had no effect on cellular viability at doses above 2,500 µM in the different cell lines (Figure 4B).

### Intracellular effects of H_2_O_2_ and Fe(II) cotreatment

Liver cells were cultured with medium (as a control), H_2_O_2_, Fe(II), or H_2_O_2_, and Fe(II) together and then charged with XTT. As indicated in Figure 4C, H_2_O_2_ lowered the mitochondrial reduction of XTT, and cell mortality increased with increasing hydrogen peroxide concentration; 880 µM H_2_O_2_ was the first dose to induce significant cell death. A similar result was found in a previous investigation by Charkoudian et al. (41); only at 2,930 µM H_2_O_2_, did cotreatment induce a decrease in cellular viability compared with H_2_O_2_ alone?

### Intracellular effects of H_2_O_2_ and Fe(III) cotreatment

The data from cotreatment with Fe(III) and various H_2_O_2_ concentrations are shown in Figure 4D. Cellular sensitivity to H_2_O_2_ coupled with Fe(III) was shown to differ significantly (*P* < 0.05) from that to H_2_O_2_ alone. The results showed that Fe(III) at non-toxic concentrations could significantly increase H_2_O_2_ toxicity, indicating the synergistic effects of H_2_O_2_ and Fe(III) in vitro. Similar to Fe(II), cytotoxicity was observed with increased concentrations of H_2_O_2_ but was more pronounced with Fe(III).

### CAT and SOD mRNA expression

**Fig. 5:**
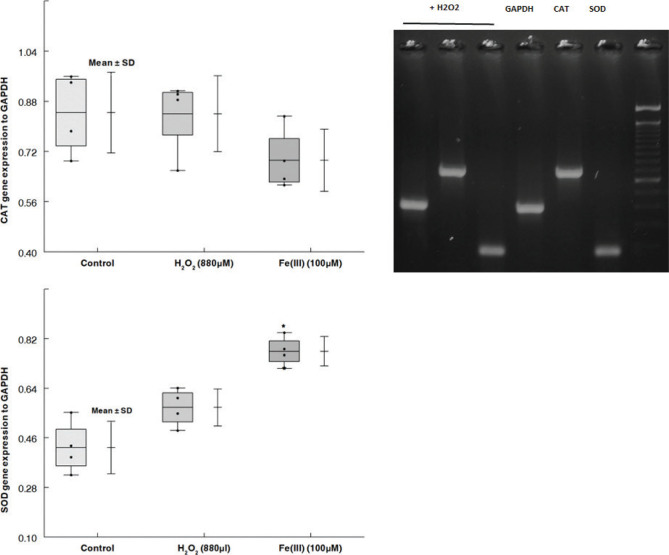
Impact of Fe(III) on SOD and CAT gene expression on FTO2B cells. RT-PCR was used to assess SOD and CAT mRNA expression. We recorded and converted gel images based on pixel value using the software UN-Scan-it V6.2 from Silk Scientific, Orem, UT, USA. Every value corresponds to mean ± SD. Using one-way ANOVA, *P < 0.05 and **P < 0.01 compared to the control.

We thought that the upregulation of the antioxidant enzymes SOD and CAT would have a protective impact if Fe(II) and Fe(III) exert their cytotoxic effects in part by oxidative stress. To evaluate this hypothesis, FTO2B cells were incubated in the presence of 880 µM H_2_O_2_ and 100 µM Fe(III). After 72 h of incubation, Fe(III) produced a significant augmentation in SOD gene expression (*P* < 0.05) compared with the control group (Figure 5). CAT and SOD mRNA levels were not significantly changed after exposure to 880 µM H_2_O_2_.

### In silico molecular docking

**Fig. 6:**
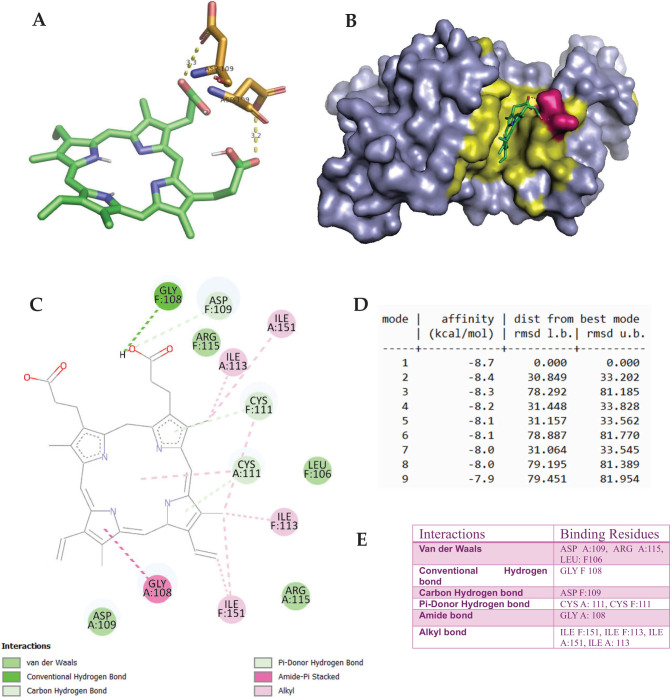
The molecular docking of heme iron (Fe^2+^-Protoporphyrin, PubChem CID: 4971) with Superoxide Dismutase (SOD, PDB ID: 2C9V). Panels A and C depict the tertiary and secondary structures of the docked complex, highlighting interactions with key target amino acid residues, respectively. Panel B presents the surface representation of the ligand-binding pocket, demonstrating the spatial orientation of heme iron within the active site. Panel D summarizes the docking scores, indicating the binding affinity and stability of the complex. Panel E illustrates specific molecular interactions, including hydrogen bonding, hydrophobic interactions, and others. Relevant ASP109 residues are distributed in different chains of the SOD dimer (PDB ID: 2C9V). The ligand corresponds to the best-docking binding pose identified from AutoDock Vina under the blind docking mode. The receptor form corresponds to the heme free tetramer.

**Fig. 7:**
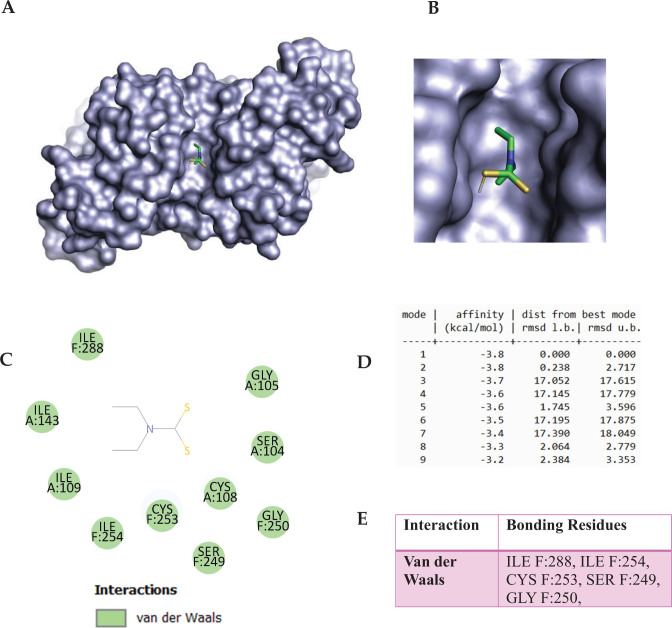
The molecular docking of Diethyldithiocarbamate (DETC) (PubChem CID: 8987) with Superoxide Dismutase (SOD, PDB ID: 2C9V). Panels A and B represent the surface representation of the ligand-binding pocket demonstrating the spatial orientation of SOD inhibitor within the active site, while Panel C represents secondary structures of the docked complex, highlighting interactions with key target amino acid residues. Panel D summarizes the docking scores, indicating the binding affinity and stability of the complex. Panel E illustrates the specific molecular interactions. The ligand corresponds to the best-docking binding pose identified from AutoDock Vina under the blind docking mode. The receptor form corresponds to the heme free tetramer.

This in silico study investigates the binding of Fe^2+^-Protoporphyrin, Diethyldithiocarbamate (DETC), and Amitrole to oxidative stress-related enzymes, SOD and CAT. Docking analysis of Fe^2+^-Protoporphyrin with SOD revealed strong binding, with a top binding energy of –8.7 kcal/mol. The docking scores ranged from –8.7 to –7.9 kcal/mol, indicating stable ligand-protein interactions (Figure 9D). Root Mean Square Deviation (RMSD) values varied significantly, with mode 1 showing a perfect alignment (0.000 Å), while other conformations exhibited flexibility up to 81.954 Å. Key molecular interactions included Van der Waals forces, hydrogen bonding, and π-donor hydrogen bonding. Hydrophobic interactions contributed to ligand stabilization within the active site (Figure 6E). DETC docking with SOD displayed a strong inhibitory effect through Van der Waals interactions (ILE F:288 and ILE F:254) and hydrophobic stabilization. DETC also formed key polar interactions (SER F:249) and engaged CYS F:253 (Figure 7).

**Fig. 8:**
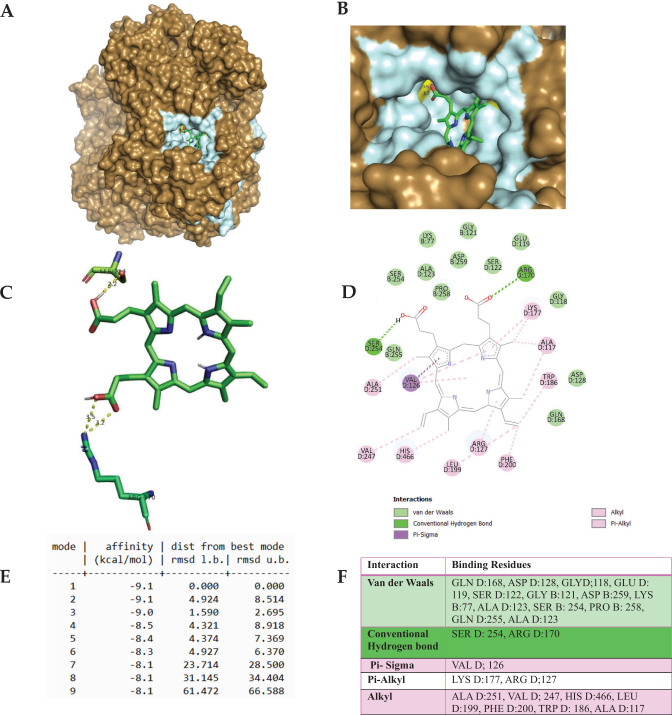
The molecular docking of heme iron (Fe^2+^-Protoporphyrin, PubChem CID: 4971) with Catalase (PDB ID: 1DGF). Panels A and B represent the surface representation of the ligand-binding pocket, demonstrating the spatial orientation of heme iron within the active site, while Panels C and D depict the tertiary and secondary structures of the docked complex, highlighting interactions with key target amino acid residues, respectively. Panel E summarizes the docking scores, indicating the binding affinity and stability of the complex. Panel F illustrates specific molecular interactions, including hydrogen bonding, hydrophobic interactions, and others. The ligand corresponds to the best-docking binding pose identified from AutoDock Vina under the blind docking mode. The receptor form corresponds to the heme free tetramer.

**Fig. 9:**
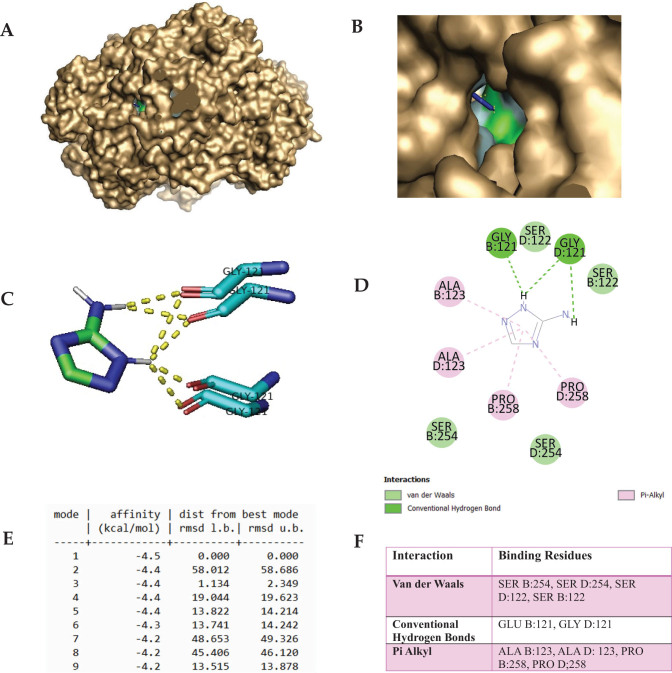
The molecular docking of Amitrole (Catalase inhibitor; 3-Amino-1,2,4-triazole (3-AT): PubChem CID: 5517) with Catalase (PDB ID: 1DGF). Panels A and B represent the surface representation of the ligand-binding pocket, demonstrating the spatial orientation of catalase inhibitor within the active site, while Panels C and D represent tertiary and secondary structure, respectively, of the docked complex. Panel E summarizes the docking scores, indicating the binding affinity and stability of the complex, while Panel F illustrates the specific molecular interactions and highlights interactions with key target amino acid residues. GLY121 residues shown represent chains A, B, C, and D of the tetrameric catalase structure (PDB ID: 1DGF). The ligand corresponds to the best-docking binding pose identified from AutoDock Vina under the blind docking mode. The receptor form corresponds to the heme free tetramer.

The docking study of heme iron (Fe^2+^-Protoporphyrin) with CAT showed a strong binding affinity with the best docking score of –9.1 kcal/mol, suggesting stable interactions. Fe^2+^-Protoporphyrin exhibited binding energy values ranging from –9.1 to –8.4 kcal/mol, with an RMSD of 0.000 Å, indicating optimal binding (Figure 8E). The docking results of Amitrole with CAT showed a moderate binding affinity of –4.5 kcal/mol in its best pose. Other docking modes had binding energies between –4.4 and –4.2 kcal/mol, with high RMSD values (up to 58.012 Å), indicating binding flexibility (Figure 9E). The docking of Amitrole in the active site of the CAT enzyme was conducted with all four chains (A, B, C, and D) of the enzyme. The analysis supports that, indeed, amitrole is bound in a similar manner with involvement of corresponding conserved residues in the active sites of chains A and C, like in chains B and D (Supplementary Figure A). This is in line with the symmetrical and homotetrameric character of CAT, whose subunit possesses an identical active site. The small differences observed for the interaction scores across chains (if there are any) are due to slight conformational discrepancies or steric hindrance stemming from the tetrameric formation. Nevertheless, these discrepancies do not appreciably alter the binding mode nor the main interacting residues. Crucially, the interacting residues all fall close to the catalytic center, which hosts the heme (protoporphyrin IX) moiety. These residues participate in either substrate access or stabilization and may also play a role in electron transfer in the catalysis. Steric hindrance of substrate entrance or changes in redox potential caused by binding of Amitrole to these sites may disturb enzymatic activity.

We subsequently completed docking analysis with a single CAT unit (monomer) (Supplementary Figure A). While in the tetrameric formation, strong binding affinities and interacting residues differ because of steric effects. This supports the stability of our results and suggests that within the isolated chain, the geometry of the active site is preserved (Supplementary Figure A and B).

## Discussion

Iron accumulates in the parenchyma of some organs, such as the heart, pancreas, liver, and endocrine organs, as a characteristic of the pathophysiology of excess iron. Iron overload causes harmful effects in rats, including hepatocellular enlargement, cardiomyopathy, pancreatic atrophy, and splenic white pulp atrophy (42). Iron is a fantastic biocatalyst but is also able to undergo opposite modifications depending on the oxidative environment, making it a potentially dangerous metal (3). Iron in the form of Fe^2+^ interacts with oxygen to produce free radicals that injure the intracellular contents, leading to cell mortality, and have been linked to iron-mediated toxicity. Because iron requires oxygen to promote free radical reactions, excess Fe^2+^ causes cellular dysfunction (42, 43). Even Fe^3+^ has often been regarded as non-cytotoxic (44, 45). In this study, intraperitoneal exposure to iron in the form of FeCl_3_ did not produce toxicologically significant modifications. The activities of AST, ALP, and ALT influenced only with the Fe^2+^ treatment, indicating the presence of hepatic injury (Figure 1). Furthermore, microscopic analysis revealed no signs of liver injury or other inflammatory processes. According to Appel et al. (46), feeding iron in the form of FeSO_4_ until 11.5 and 11.2 mg/kg body weight/day had no toxicologically significant consequences and did not cause tissue iron overload.

Oxygen-free radicals are scavenged by SOD and CAT, which may be indirect indicators of the body’s antioxidant capacity (47). The final product of lipid peroxidation (MDA) is generally used as a bioindicator of lipid peroxidation, is associated with the intensity of the pertinent-free radical response, and indirectly assesses the level of hepatocyte damage (48–50). Our *in vivo* results revealed that the activities of SOD and CAT were augmented in response to ROS production, as revealed by the increase in MDA content (Figure 2). In general, the liver is exposed to too much iron more often than other organs because it is the primary and main organ for iron storage (26). Indeed, our data highlight that Fe^2+^ at levels normally found in many species of Red Sea fish could pose a risk to human health.

In the *in vitro* study, we evaluated the oxidant effect of iron in FTO_2_B cells. In particular, we assessed the involvement of transition metal ions (Fe^2+^ and Fe^3+^) in the oxidative stress environment already induced by extracellular H_2_O_2_ on cell viability. H_2_O_2_ is thought to have the ability to effortlessly penetrate biological membranes and diffuse a great distance from its source since it is an uncharged molecule with a low molecular mass (51–53). High levels of H_2_O_2_ (more than 50 µM) have often been found to be toxic to many animals, plants, and bacterial cells, which are consistent with our results. Indeed, several factors influence how cells die, such as the cell line used, length of exposure to H_2_O_2_, iron content, concentration of H_2_O_2_ employed, and cell culture medium used (21, 54, 55). In the current work, we found evidence Fe^2+^ toxicity to only FTO_2_B cells at high concentrations (2,500 µM), and the experimental setup revealed no cell death after Fe(III) treatment. However, the results showed that Fe(III) at a non-toxic concentration (100 µM) significantly increased H_2_O_2_ toxicity and markedly augmented cell death above the level caused by H_2_O_2_ alone, indicating a synergistic effect between H_2_O_2_ and Fe(III). When cotreated with H_2_O_2_, there seems to be cytotoxicity from Fe^3+^ and no effect or a protection from Fe^2+^. Chamnongpol et al. (3) reported that Fe^3+^ can induce cellular toxicity, and that Fe^3+^ mediates its cytotoxicity via a process that is oxygen independent but unlike the process induced by Fe^2+^. Zhao et al. (56) establish that Fe^3+^ performs biphasic roles in cultured porcine parthenotes. They showed that redundant Fe^3+^ conducted to high ROS concentration, lowering that redundant Fe^3+^ decreased ROS content, and mitochondrial function is further protected, although excessive Fe^3+^ depletion reduced mitochondrial function, resulting in blastocyst apoptosis. According to Leiter et al. (57), concentrations between 150 and 300 µmol/L of extracellular Fe^3+^ increased the oxidative stress caused by 700 mmol/L H_2_O_2_ when fungi cultured and grown in complex medium. We hypothesized that cotreating cells with H_2_O_2_ plus Fe^2+^ can increase cell injury and consequently cell death. However, to our surprise, the incorporation of Fe^2+^ did not change the harmful effects of H_2_O_2_. This finding is consistent with the outcome provided by Hempel et al. (58), who established that extracellular Fe(II) can defend cells against hydrogen peroxide-induced injury and speculated that extracellular Fe(II) initiates the Fenton reaction on the exterior of the cells where a large amount of hydroxyl radicals (HO^•^) react with medium components, safeguarding the interior cellular environment from H_2_O_2_ (58). According to the authors, the lack of intracellular damage caused by H_2_O_2_ and Fe^2+^ supports the idea that most or all the hazardous signals are produced outside of the cell. Additionally, exposure to Fe(III) enhanced SOD mRNA expression. There was plenty of *in vitro* evidence of cellular damage, including damage from HO^•^, when these antioxidants are unable to limit extracellular oxidant generation (52). Furthermore, the apparent Fe(III)-induced expression of antioxidant genes suggests that iron might serve as a physiological signal that mediates the cellular reaction to oxidative stress caused by H_2_O_2_.

Docking analysis of Fe^2+^-Protoporphyrin with SOD revealed strong binding, with a top binding energy of –8.7 kcal/mol. The docking scores ranged from –8.7 to –7.9 kcal/mol, indicating stable ligand–protein interactions. RMSD values varied significantly, with mode 1 showing a perfect alignment (0.000 Å), while other conformations exhibited flexibility up to 81.954 Å (Figure 9D). Key molecular interactions included Van der Waals forces (ASP A:109 and ARG A:115), hydrogen bonding (GLY F:108 and ASP F:109), and π-donor hydrogen bonding (CYS A:111 and CYS F:111). Hydrophobic interactions (ILE F:151, ILE F:113, and ILE A:151) contributed to ligand stabilization within the active site (Figure 9E). Comparative literature analysis suggests that Fe^2+^ binding may interfere with enzymatic activity, similar to studies on metal ion displacement in SOD (59). Research by Dai et al. further supports the role of Fe^2+^ in oxidative enzyme inhibition, which aligns with the observed docking interactions (60).

Docking of DETC with SOD yielded a binding affinity of -3.8 kcal/mol, suggesting a relatively weak interaction under the docking conditions used. Although DETC is known as an SOD inhibitor in experimental studies, the current docking result alone does not strongly support this mechanism. Additionally, in vitro or comparative docking studies with known SOD inhibitors would be necessary to validate its inhibitory potential. DETC also formed key polar interactions (SER F:249) and engaged CYS F:253, which may contribute to its known metal-chelating properties (Figure 10E). Literature confirms DETC as an SOD inhibitor via Cu/Zn ion sequestration (61–64). DETC inhibition of SOD has been linked to oxidative stress and apoptosis, reinforcing its potential role in neurodegenerative disorders. The biological implications of these findings highlight the role of Fe^2+^ and DETC in oxidative stress modulation. Overall, both Fe^2+^-Protoporphyrin and DETC exhibit strong interactions with oxidative stress enzymes, with Fe^2+^ potentially enhancing oxidative damage and DETC functioning as an effective inhibitor through metal ion sequestration.

On the other hand, the docking study of heme iron (Fe^2+^-Protoporphyrin) with CAT showed a strong binding affinity with the lowest docking score of –9.1 kcal/mol, suggesting stable interactions. Fe^2+^-Protoporphyrin exhibited binding energy values ranging from –9.1 to –8.4 kcal/mol, with an RMSD of 0.000 Å, indicating optimal binding (Figure 11E). Key Van der Waals interactions were observed with residues HIS A:75, ASP A:181, and GLU A:351, stabilizing the ligand in the active site. Hydrogen bonding interactions were detected with ARG A:354 and ASN A:148, enhancing binding specificity. Pi-Pi stacking with TYR A:358 and hydrophobic interactions with LEU A:144 and PHE A:161 further contributed to ligand retention (Figure 11F). Previous study showed that heme iron can inhibit CAT activity, potentially leading to oxidative stress by interfering with H_2_O_2_ breakdown enhancing ROS formation (65). Additional molecular dynamics simulations and experimental assays are needed to validate these inhibitory effects.

The docking study of Amitrole with CAT showed a moderate binding affinity of –4.5 kcal/mol in its best pose. Other docking modes had binding energies between –4.4 and –4.2 kcal/mol, with high RMSD values (up to 58.012 Å), indicating binding flexibility (Figure 12E). Van der Waals interactions involved SER B:254, SER D:254, SER D:122, and SER B:122, contributing to ligand stability. Hydrogen bonds were formed with GLU B:121 and GLY D:121, enhancing specificity. Pi-Alkyl interactions with ALA B:123, ALA D:123, PRO B:258, and PRO D:258 provided additional stabilization. Despite moderate binding, Amitrole’s high binding flexibility suggests potential instability in physiological conditions. Compared to Fe^2+^-Protoporphyrin, Amitrole binds less tightly (–4.5 kcal/mol vs. –9.1 kcal/mol), making Fe^2+^ a stronger and potentially more disruptive inhibitor of CAT. Heme iron binds more strongly to CAT than Amitrole, indicating a higher potential to inhibit enzymatic function and contribute to oxidative stress. Amitrole exhibits moderate binding affinity but high RMSD values, suggesting weaker and less stable interactions compared to Fe^2+^-Protoporphyrin. While both compounds could act as CAT inhibitors, heme iron is the more potent inhibitor based on its stronger and more stable binding.

## Conclusions

The in vivo and in vitro observations in this study indicated that cells must be stressed after consuming moderate amounts of Fe(II) and Fe(III) from fish products. The docking analysis revealed that Fe^2+^-Protoporphyrin exhibited strong binding to both SOD (-8.7 kcal/mol) and CAT (–9.1 kcal/mol), suggesting a potential role in enzymatic inhibition and oxidative stress modulation. Thus, information on iron and/or metal concentrations in fish and sea products in general is important to assess the possible exposure of the community to toxic compounds after their consumption. Consequently, the determination of the estimated weekly intake of iron by people consuming different species of fish will be significant for human health and safety.

## Supplementary Material


