# Supplementary material for: Impact of environmental contaminants in fish on cell death and oxidative stress using in vivo, in vitro, and molecular docking
Source: Food Nutr Res. 2025 Oct 28;69:10.29219/fnr.v69.12687. doi: 10.29219/fnr.v69.12687 (PMC12581650; doi:10.29219/fnr.v69.12687)
Supplement: Supplementary file 1 [file FNR-69-12687-s1.docx]

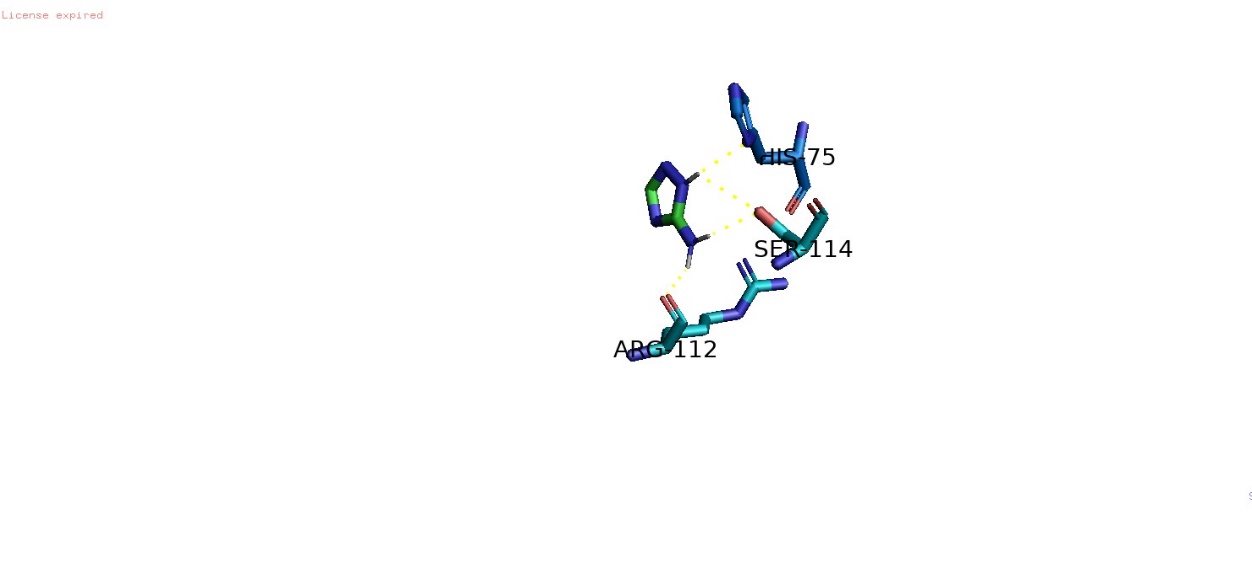

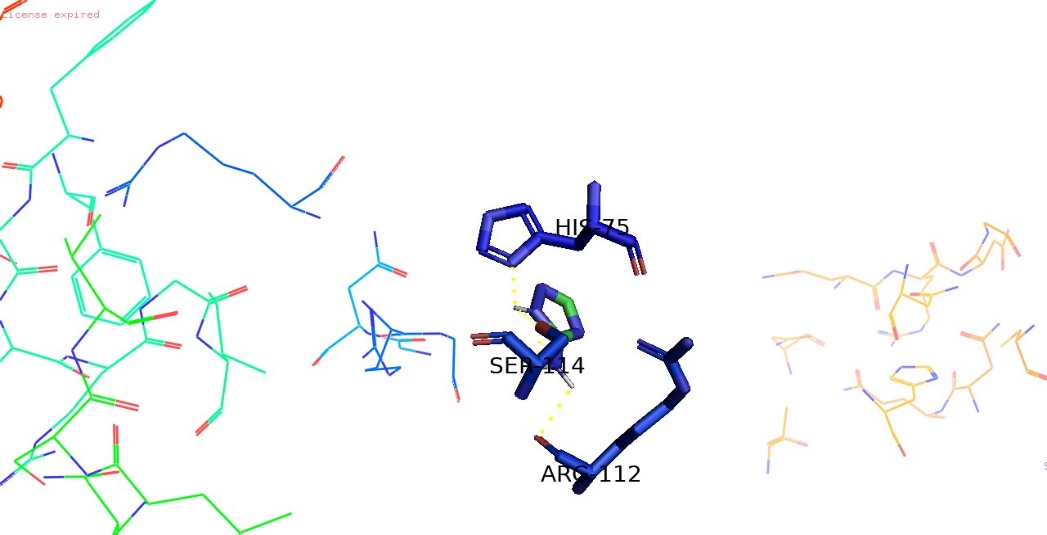

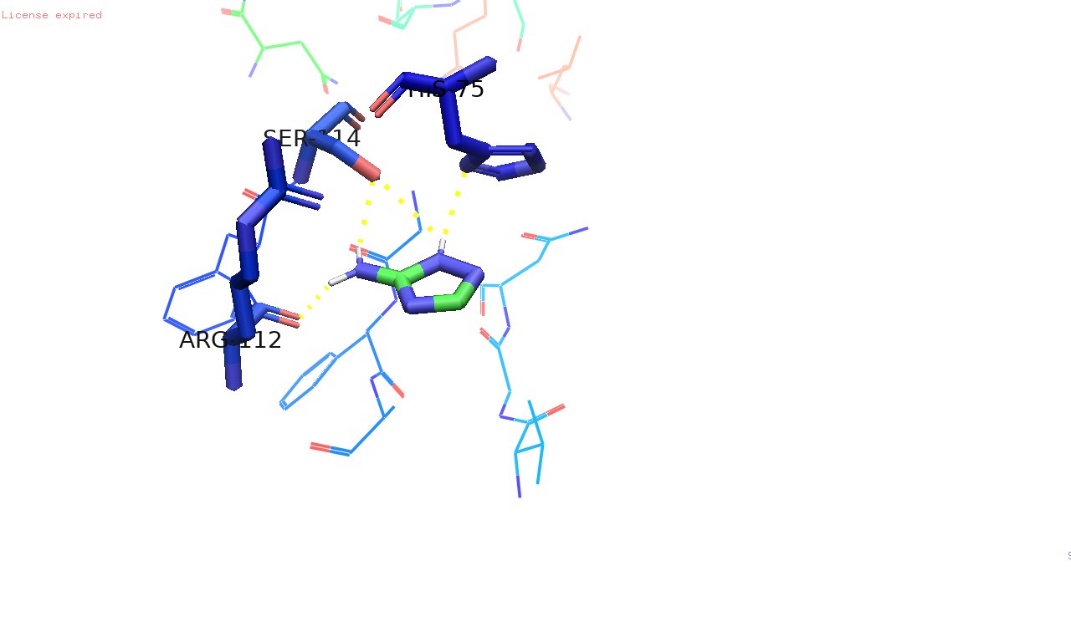

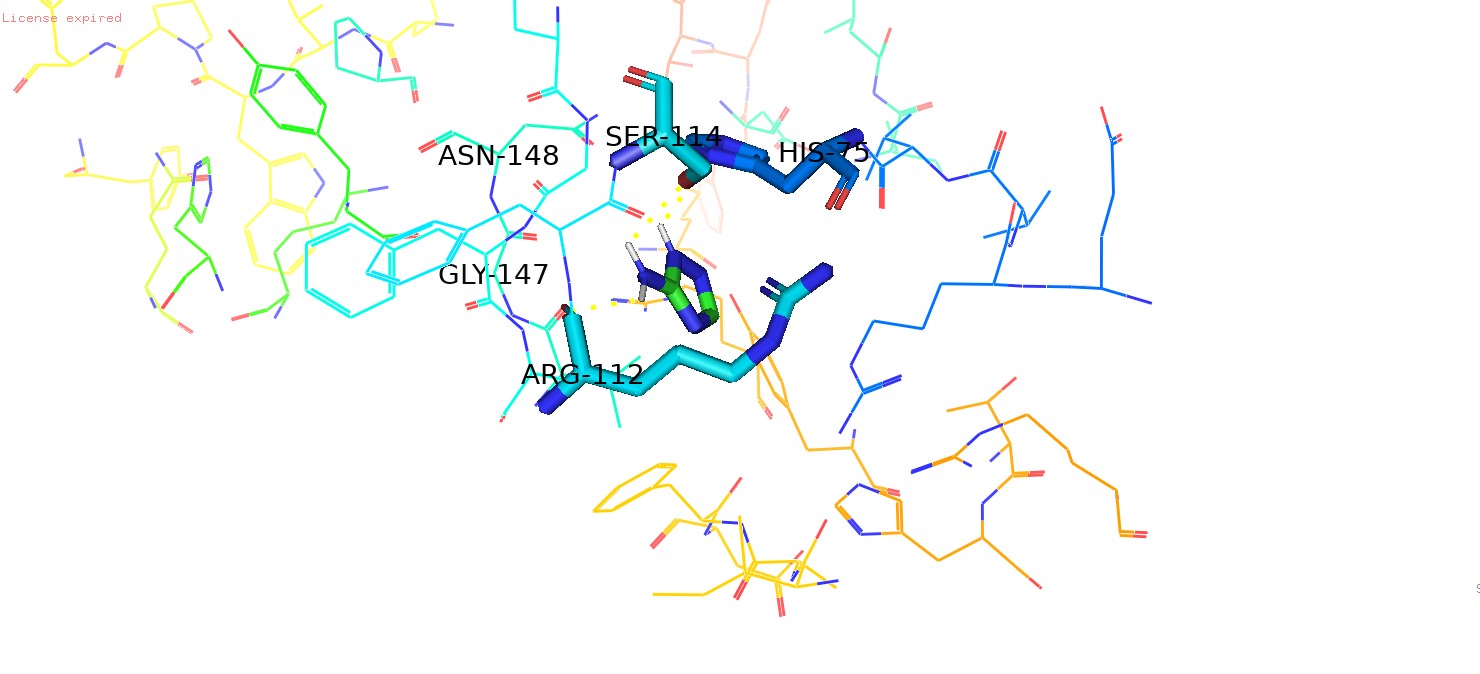


**Chain A**

**Chain B**

**Chain C**

**Chain D**

**Figure A:** Amitrole showing similar binding patterns of HIS-75, SER-114 and ARG-112 across all chains, interacting with corresponding conserved residues in the active sites of chains A and C as it does with chains B and D.


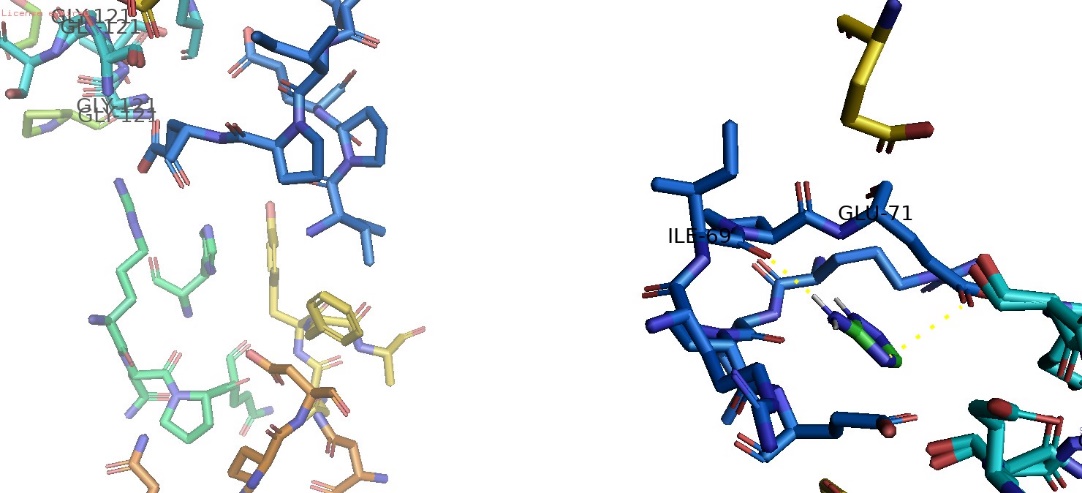

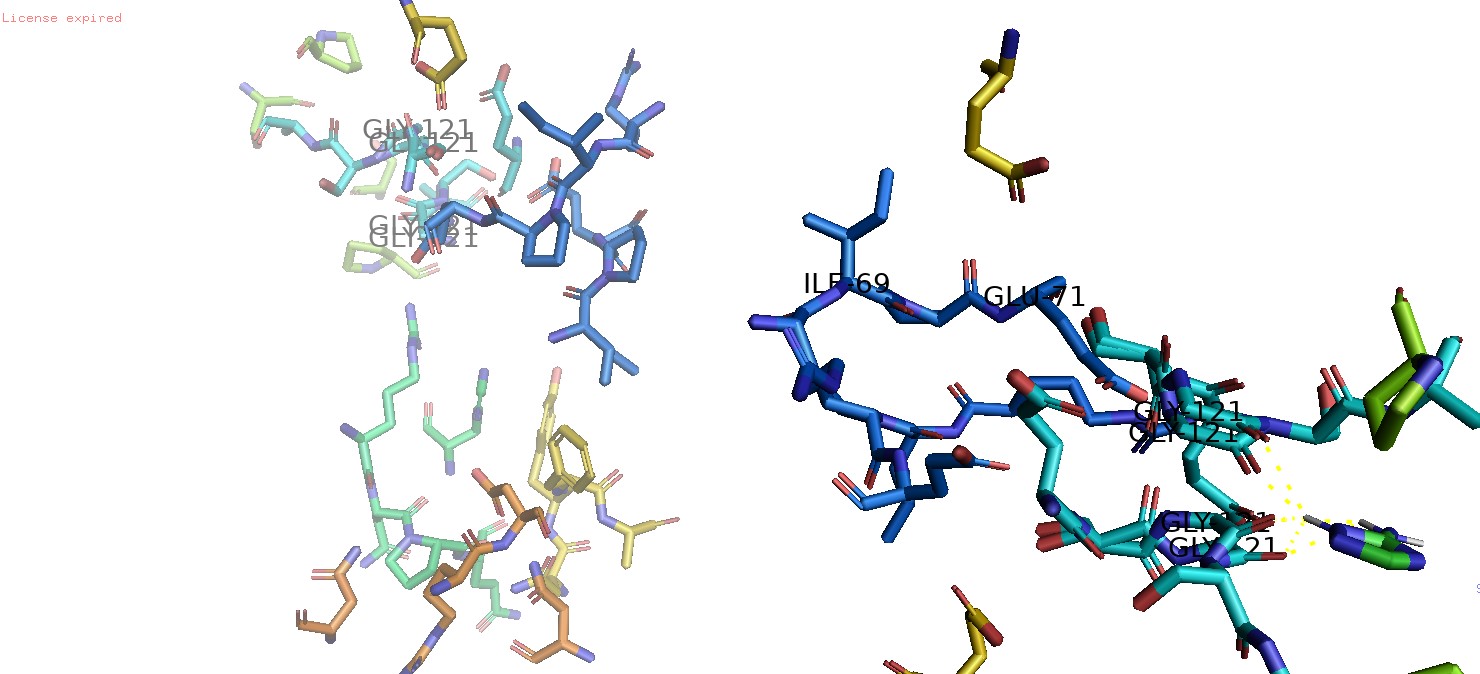


**Figure B:** Amitrole showing similar binding patterns in tetrameric chains. On docking first position Amitrole binds with ILE-69 and GLU-71 and on the 2^nd^ position binds with GLY121.
